# Poly[diamminedi-μ_3_-dicyanamido-copper(II)]

**DOI:** 10.1107/S1600536812045382

**Published:** 2012-11-10

**Authors:** Jesús García Díaz, Atzimba García Albor, Espino Valencia Jaime, Viktor Vrábel, Jozef Kožíšek

**Affiliations:** a Faculty Chemistry Engineering, Michoacán University, Morelia, Michoacán, Mexico; b COFEPRIS, Michoacán University, Morelia, Michoacán, Mexico; c Institute of Analytical Chemistry, Faculty of Chemical and Food Technology, Slovak University of Technology, Radlinského 9, SK-812 37 Bratislava, Slovak Republic 81237; d Institute of Physical Chemistry and Chemical Physics, Slovak University of Technology, Radlinského 9, SK-812 37 Bratislava, Slovak Republic

## Abstract

The asymmetric unit of the title polymeric mononuclear Cu^II^ complex, [Cu(C_2_N_3_)_2_(NH_3_)_2_]_*n*_, contains one half-mol­ecule, the complex being completed through inversion symmetry, with the Cu^II^ atom situated on the centre of symmetry. The coordination polyhedron around Cu^II^ is a Jahn–Teller-distorted [CuN_6_] octa­hedron. The terminal N atoms of two dicyanamide ligands and two ammine ligands form an approximate square plane, with N—Cu—N bite angles of 89.72 (5) and 90.28 (5)°. The coordination polyhedron is completed in the axial positions by the central amide-type N atoms of two additional dicyanamide ligands, with an elongated Cu—N distance of 2.548 (1) Å. In turn, each of the four dicyanamide ligands, acting as bidentate, link the Cu^II^ ions into a two-dimensional polymeric structure parallel to (100). The ammine H atoms are involved in inter­molecular hydrogen bonding with the free terminal N atoms of neighbouring dicyanamide ligands, yielding a three-dimensional network.

## Related literature

For bonding modes of the dicyanamide ligand, see: Burčák *et al.* (2004 ▶); Yang *et al.* (2004 ▶); van Albada *et al.* (2001 ▶); Potočňák *et al.* (2002 ▶); Zhang *et al.* (2004 ▶); Mohamadou *et al.* (2003 ▶); Batten *et al.* (2000 ▶); Kožíšek *et al.* (2007 ▶). For magnetic properties of [*M*(dicyanamide)_2_] compounds, see: Batten & Murray (2003 ▶); Kurmoo & Kepert (1998 ▶).
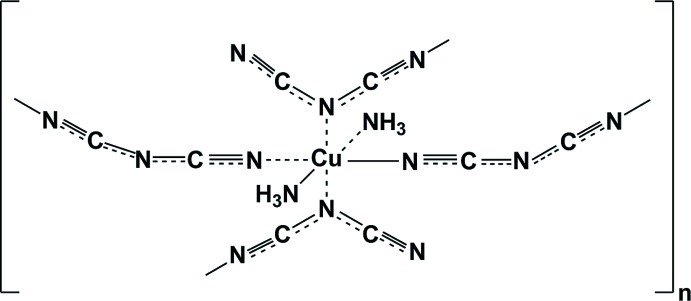


## Experimental

### 

#### Crystal data

[Cu(C_2_N_3_)_2_(NH_3_)_2_]
*M* *_r_* = 229.72
Monoclinic, 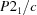

*a* = 7.1310 (2) Å
*b* = 9.6301 (2) Å
*c* = 7.2162 (2) Å
β = 113.782 (3)°
*V* = 453.47 (2) Å^3^
*Z* = 2
Mo *K*α radiation
μ = 2.38 mm^−1^
*T* = 298 K
0.52 × 0.32 × 0.17 mm

#### Data collection

Oxford Diffraction Gemini R CCD diffractometer
Absorption correction: analytical [*CrysAlis RED* (Oxford Diffraction, 2010) ▶, based on expressions derived by Clark & Reid (1995 ▶)] *T*_min_ = 0.410, *T*_max_ = 0.682
19836 measured reflections
1126 independent reflections
1019 reflections with *I* > 2σ(*I*)
*R*_int_ = 0.016

#### Refinement

*R*[*F* ^2^ > 2σ(*F* ^2^)] = 0.017
*wR*(*F* ^2^) = 0.052
*S* = 1.07
1126 reflections
74 parameters
H atoms treated by a mixture of independent and constrained refinement
Δρ_max_ = 0.21 e Å^−3^
Δρ_min_ = −0.25 e Å^−3^

### 

Data collection: *CrysAlis CCD* (Oxford Diffraction, 2010 ▶); cell refinement: *CrysAlis CCD*; data reduction: *CrysAlis RED* (Oxford Diffraction, 2010 ▶); program(s) used to solve structure: *SHELXS97* (Sheldrick, 2008 ▶); program(s) used to refine structure: *SHELXL97* (Sheldrick, 2008 ▶); molecular graphics: *DIAMOND* (Brandenburg, 1998 ▶); software used to prepare material for publication: *enCIFer* (Allen *et al.*, 2004 ▶).

## Supplementary Material

Crystal structure: contains datablock(s) I, global. DOI: 10.1107/S1600536812045382/lr2087sup1.cif

Structure factors: contains datablock(s) I. DOI: 10.1107/S1600536812045382/lr2087Isup2.hkl

Additional supplementary materials:  crystallographic information; 3D view; checkCIF report

## Figures and Tables

**Table 1:** Hydrogen-bond geometry (Å, °)

*D*—H⋯*A*	*D*—H	H⋯*A*	*D*⋯*A*	*D*—H⋯*A*
N1—H1⋯N4^i^	0.84 (2)	2.43 (2)	3.2555 (18)	165.4 (19)
N1—H2⋯N4^ii^	0.89 (2)	2.34 (2)	3.2278 (18)	175.7 (17)
N1—H3⋯N4^iii^	0.81 (2)	2.43 (2)	3.2073 (18)	162.1 (19)

Symmetry codes: (i) 
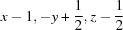
; (ii) 
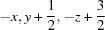
; (iii) 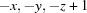.

**Fig. 1.**
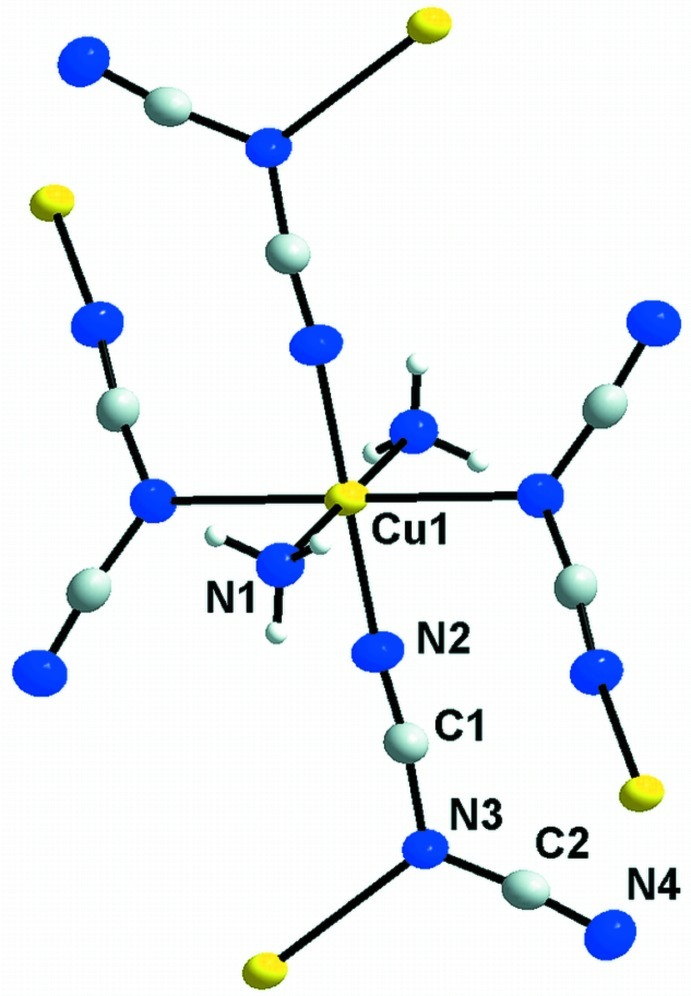
Part of the polymeric structure of (I), with the displacement ellipsoids drawn at the 50% probability level.

**Fig. 2.**
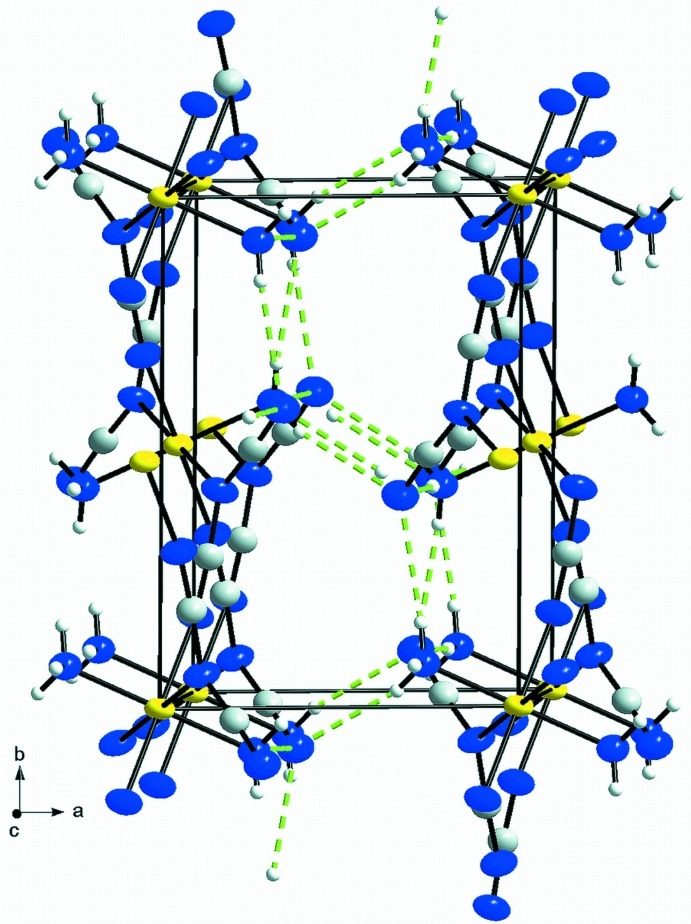
A packing diagram for (I) showing the hydrogen-bonding network as green dashed lines.

## supplementary crystallographic information

### Comment

Among the various classes of ligands currently employed for the generation of coordination compounds, dicyanamide (dca) has been attracting a lot of attention, partly due to the discovery of interesting magnetic properties in the *M*(dca)_2_ compounds (Batten & Murray, 2003; Kurmoo & Kepert, 1998). A particular feature of this ligand is the variability in coordination modes it can display and thus it is able to generate one- to three-dimensional networks, as well as molecular and ionic compounds, depending on its metallic centers and its organic coligands. In coordination compounds of copper, the dca anion, (N(CN)_2_), exhibits a rich variety of bonding modes. It can coordinate either in a monodentate manner (Burčák *et al.*, 2004; Yang *et al.*, 2004;) or, more typically, in a bidentate manner [two types of binding: mainly through two nitrile N atoms (Albada *et al.*, 2001; Potočňák *et al.*, 2002; Zhang *et al.*, 2004), but also through one amide and one nitrile N atom (Mohamadou *et al.*, 2003), or even in a tridentate manner (Batten *et al.*, 2000; Kožíšek *et al.*, 2007). The asymmetric unit of the title compound, (I), [Cu(N(CN)_2_)_2_(NH_3_)_2_]_n_, contains one-half of the molecule with the Cu^II^ atom situated at the centre of symmetry and is octahedrally coordinated by two ammino and two bidentate dca ligands, forming a CuN6 coordination environment (Fig.1). Two terminal N atoms of two dca units and two ammino ligands forming an approximate square plane with N—Cu—N bite angles of 89.72 (5) and 90.28 (5)°. Coordinaton polyhedron is completed in axial position by the central amide N atoms of two additional dca ligands with the Cu–N elongated distance of 2.548 (1) Å as a result of the Jahn–Teller effect. The amino H atoms are involved in intermolecular hydrogen bonding with the free terminal N atoms of neighbouring dicyanamide ligands, yielding a three-dimensional network (Fig.2).

### Experimental

A solution of Cu(SO_4_)_2_.5H_2_O (2.0 mmol) in water (3 ml) was added to a solution of K[N(CN)_2_] (4.0 mmol) in water (10 ml)and mixed with a solution of ammine (4.0 mmol) in water (10 ml). After standing for a few days, blue crystals of (I) were isolated (yield: *ca* 10%).

### Refinement

The ammine H atoms were located in a difference Fourier map and refined with a fixed isotropic displacement parameter.

### Crystal data

**Table:**

[Cu(C_2_N_3_)_2_(NH_3_)_2_]	*F*(000) = 230
*M**_r_* = 229.72	*D*_x_ = 1.682 Mg m^−^^3^
Monoclinic, *P*2_1_/*c*	Mo *K*α radiation, λ = 0.71073 Å
Hall symbol: -P 2ybc	Cell parameters from 15460 reflections
*a* = 7.1310 (2) Å	θ = 3.7–29.3°
*b* = 9.6301 (2) Å	µ = 2.38 mm^−^^1^
*c* = 7.2162 (2) Å	*T* = 298 K
β = 113.782 (3)°	Block, dark blue
*V* = 453.47 (2) Å^3^	0.52 × 0.32 × 0.17 mm
*Z* = 2	

### Data collection

**Table:**

Oxford Diffraction Gemini R CCD diffractometer	1126 independent reflections
Radiation source: fine-focus sealed tube	1019 reflections with *I* > 2σ(*I*)
Graphite monochromator	*R*_int_ = 0.016
Detector resolution: 10.4340 pixels mm^-1^	θ_max_ = 28.3°, θ_min_ = 3.7°
ω and φ scans	*h* = −9→9
Absorption correction: analytical [*CrysAlis RED* (Oxford Diffraction, 2010), based on expressions derived by Clark & Reid (1995)]	*k* = −12→12
*T*_min_ = 0.410, *T*_max_ = 0.682	*l* = −9→9
19836 measured reflections	

### Refinement

**Table:**

Refinement on *F*^2^	Primary atom site location: structure-invariant direct methods
Least-squares matrix: full	Secondary atom site location: difference Fourier map
*R*[*F*^2^ > 2σ(*F*^2^)] = 0.017	Hydrogen site location: inferred from neighbouring sites
*wR*(*F*^2^) = 0.052	H atoms treated by a mixture of independent and constrained refinement
*S* = 1.07	*w* = 1/[σ^2^(*F*_o_^2^) + (0.0314*P*)^2^ + 0.113*P*] where *P* = (*F*_o_^2^ + 2*F*_c_^2^)/3
1126 reflections	(Δ/σ)_max_ < 0.001
74 parameters	Δρ_max_ = 0.21 e Å^−^^3^
0 restraints	Δρ_min_ = −0.25 e Å^−^^3^

### Special details

**Table:**

Experimental. face-indexed (*CrysAlis RED*; Oxford Diffraction, 2010)Absorption correction: CrysAlis RED, Oxford Diffraction Ltd., Version 1.171.32.8 (release 30-07-2007 CrysAlis171 .NET) (compiled Jul 30 2007,18:35:48) Analytical numeric absorption correction using a multifaceted crystal model based on expressions derived by R.C. Clark & J.S. Reid. (Clark, R. C. & Reid, J. S. (1995). Acta Cryst. A51, 887-897)
Geometry. All e.s.d.'s (except the e.s.d. in the dihedral angle between two l.s. planes) are estimated using the full covariance matrix. The cell e.s.d.'s are taken into account individually in the estimation of e.s.d.'s in distances, angles and torsion angles; correlations between e.s.d.'s in cell parameters are only used when they are defined by crystal symmetry. An approximate (isotropic) treatment of cell e.s.d.'s is used for estimating e.s.d.'s involving l.s. planes.
Refinement. Refinement of *F*^2^ against ALL reflections. The weighted *R*-factor *wR* and goodness of fit *S* are based on *F*^2^, conventional *R*-factors *R* are based on *F*, with *F* set to zero for negative *F*^2^. The threshold expression of *F*^2^ > σ(*F*^2^) is used only for calculating *R*-factors(gt) *etc*. and is not relevant to the choice of reflections for refinement. *R*-factors based on *F*^2^ are statistically about twice as large as those based on *F*, and *R*- factors based on ALL data will be even larger.

### Fractional atomic coordinates and isotropic or equivalent isotropic displacement parameters (Å^2^)

**Table:**

	*x*	*y*	*z*	*U*_iso_*/*U*_eq_	
C1	0.12500 (18)	0.20615 (12)	0.40737 (17)	0.0314 (2)	
C2	0.2567 (2)	−0.00704 (11)	0.5168 (2)	0.0313 (3)	
N1	−0.26849 (18)	0.41648 (13)	0.45514 (19)	0.0362 (2)	
H1	−0.351 (3)	0.472 (2)	0.371 (4)	0.054 (6)*	
H2	−0.283 (3)	0.417 (2)	0.572 (3)	0.053 (5)*	
H3	−0.281 (3)	0.338 (3)	0.410 (3)	0.062 (6)*	
N2	0.09027 (19)	0.32110 (12)	0.42058 (18)	0.0416 (3)	
N3	0.15107 (18)	0.07772 (11)	0.36879 (16)	0.0389 (3)	
N4	0.3460 (2)	−0.09051 (13)	0.63406 (19)	0.0462 (3)	
Cu1	0.0000	0.5000	0.5000	0.02839 (10)	

### Atomic displacement parameters (Å^2^)

**Table:**

	*U*^11^	*U*^22^	*U*^33^	*U*^12^	*U*^13^	*U*^23^
C1	0.0349 (5)	0.0285 (6)	0.0293 (5)	0.0008 (4)	0.0114 (4)	−0.0034 (4)
C2	0.0340 (6)	0.0271 (6)	0.0332 (6)	−0.0019 (4)	0.0140 (5)	−0.0056 (4)
N1	0.0405 (6)	0.0299 (5)	0.0365 (6)	−0.0005 (4)	0.0138 (5)	−0.0001 (5)
N2	0.0518 (7)	0.0276 (5)	0.0431 (6)	0.0054 (5)	0.0168 (5)	−0.0042 (4)
N3	0.0520 (6)	0.0267 (5)	0.0313 (5)	0.0077 (4)	0.0097 (5)	−0.0046 (4)
N4	0.0520 (7)	0.0353 (6)	0.0459 (6)	0.0033 (5)	0.0140 (5)	0.0045 (5)
Cu1	0.03572 (14)	0.01832 (13)	0.03050 (14)	0.00135 (6)	0.01271 (10)	−0.00132 (6)

### Geometric parameters (Å, º)

**Table:**

C1—N2	1.1466 (17)	N1—H2	0.89 (2)
C1—N3	1.2975 (16)	N1—H3	0.81 (2)
C2—N4	1.1546 (18)	N2—Cu1	2.0021 (11)
C2—N3	1.3135 (17)	Cu1—N1^i^	1.9793 (12)
N1—Cu1	1.9793 (12)	Cu1—N2^i^	2.0021 (11)
N1—H1	0.84 (2)		
			
N2—C1—N3	172.98 (14)	C1—N2—Cu1	163.67 (11)
N4—C2—N3	173.97 (14)	C1—N3—C2	120.18 (11)
Cu1—N1—H1	102.1 (15)	N1^i^—Cu1—N1	180.00 (7)
Cu1—N1—H2	108.6 (12)	N1^i^—Cu1—N2	89.72 (5)
H1—N1—H2	111 (2)	N1—Cu1—N2	90.28 (5)
Cu1—N1—H3	112.9 (15)	N1^i^—Cu1—N2^i^	90.28 (5)
H1—N1—H3	112 (2)	N1—Cu1—N2^i^	89.72 (5)
H2—N1—H3	110.2 (19)	N2—Cu1—N2^i^	180.0
			
N3—C1—N2—Cu1	124.8 (10)	C1—N2—Cu1—N1^i^	130.8 (4)
N2—C1—N3—C2	−178.5 (11)	C1—N2—Cu1—N1	−49.2 (4)
N4—C2—N3—C1	179 (100)	C1—N2—Cu1—N2^i^	24 (100)

Symmetry code: (i) −*x*, −*y*+1, −*z*+1.

### Hydrogen-bond geometry (Å, º)

**Table:**

*D*—H···*A*	*D*—H	H···*A*	*D*···*A*	*D*—H···*A*
N1—H1···N4^ii^	0.84 (2)	2.43 (2)	3.2555 (18)	165.4 (19)
N1—H2···N4^iii^	0.89 (2)	2.34 (2)	3.2278 (18)	175.7 (17)
N1—H3···N4^iv^	0.81 (2)	2.43 (2)	3.2073 (18)	162.1 (19)

Symmetry codes: (ii) *x*−1, −*y*+1/2, *z*−1/2; (iii) −*x*, *y*+1/2, −*z*+3/2; (iv) −*x*, −*y*, −*z*+1.
